# Holding on to tradition: continuation of ethnic and festival food traditions by Nepalese students in the United States

**DOI:** 10.3389/fsoc.2026.1870335

**Published:** 2026-07-07

**Authors:** Anu Karki, Shuyang Qu, Fally Masambuka-Kanchewa, Daniela Dimitrova

**Affiliations:** 1 Department of Agricultural Education and Studies, College of Agriculture and Life Sciences, Iowa State University, Ames, IA, United States; 2 Greenlee School of Journalism and Communication, College of Liberal Arts and Sciences, Iowa State University, Ames, IA, United States

**Keywords:** international students, food and culture, Nepalese students, ethnic foods, festival foods, food acculturation, cultural identity and wellbeing

## Abstract

**Introduction:**

Traditional foodways can help international students preserve cultural continuity and cope emotionally and socially in a new environment, while limited access to traditional foods leads to cultural disconnection and a negative impact on mental wellbeing. This study explored what ethnic and festival foods and practices Nepalese international students retain in the U.S., influencing factors, and the perceived significance of sustaining these practices.

**Methods:**

A qualitative phenomenological approach was employed in the present study. Three focus groups and 18 virtual interviews were conducted with 36 Nepalese international students enrolled in universities across the U.S. Midwest, guided by a semi-structured interview protocol. Thematic analysis was used to analyze the de-identified data transcripts. Data was analyzed inductively using MAXQDA Analytics Pro 24. Trustworthiness was ensured through triangulation, member-checking, audit trails and pilot-testing.

**Results:**

Six themes emerged from the participants' responses: 1) ethnic and festival foods on the table, 2) selective continuation of religious fasting practices, 3) communal celebration, 4) barriers to continuation, 5) related memories, and 6) emotional resonance through ethnic and festival foods. Findings revealed that Nepalese students demonstrated cultural resilience and agency by continuing major ethnic and festival foods. While communal gatherings and social networks facilitated these practices, continuation was limited by barriers such as time constraints, lack of culinary skills, and ingredient availability. Fasting and rituals were selectively maintained, often influenced by familial reminders and social connections. Memories and emotional ties to traditional foods reinforced the importance of traditional foods in sustaining cultural identity and psychological wellbeing.

**Discussion:**

Ultimately, Nepalese students' engagement with traditional foodways reflected a dynamic negotiation between cultural preservation and adaptation, revealing how food traditions endure, evolve, or recede based on context and capacity. These findings contribute to broader discourses in food acculturation and international education, highlighting the vital role of cultural foods in fostering cultural and emotional wellbeing among international students.

## 1 Introduction

In the context of globalization, international education has become a popular avenue for mobility, with a significant number of Nepalese youths seeking academic and professional advancement abroad (Vaidya et al., 2024). In the 2024–2025 academic year, Nepal ranked sixth among leading countries of origin for international students in the U.S. higher education (Institute of International Education, 2025a). Reflecting a 48.7% increase from the previous year, a total of 24,890 Nepalese students were enrolled in U.S. institutions in 2024–25. Managing academic obligations alongside work commitments is found to be a challenging feat for Nepalese students pursuing higher education in the U.S., as they navigate cultural adjustment within a complex foreign environment shaped by intersecting cultural, economic, and social factors (Vaidya et al., 2024).

International students frequently undergo dietary acculturation, a dynamic process through which they adapt to the food practices of the host country while also preserving aspects of their traditional diet (Shi et al., 2021). Food, beyond its nutritional function, is a powerful expression of cultural and ethnic identity, embodied through traditional foodways such as preparation, sharing, and communal consumption (Warde, 1994; Wright et al., 2021). These foodways serve as both tangible and symbolic practices that help immigrants and international students maintain cultural continuity and navigate the emotional and social adjustments of migration (D'Sylva and Beagan, 2011). Due to the deep connection between food and identity, they often make deliberate efforts to preserve their culinary traditions in unfamiliar environments (Boutaud et al., 2016). Such practices can evoke comforting memories of home, family, and belonging, offering emotional and psychological relief (Abarca and Colby, 2016). For those unable to return home, consuming traditional foods becomes a crucial way to sustain transnational ties, reaffirm cultural identity, and maintain a familiar sense of self (Wright et al., 2021).

Limited access to culturally familiar foods has been found to adversely affect the mental wellbeing of international students, contributing to feelings of stress, isolation, and cultural disconnection during the dietary transition in the host country (Alakaam and Willyard, 2020; Shi et al., 2021). The decline in dietary diversity and the shift away from culturally rooted food practices in host countries affect not only individual health but also threaten cultural sustainability, making it increasingly difficult to preserve traditional diets and the cultural identities they embody (Wright et al., 2021). Thus, gaining insight into the maintenance of traditional food practices among international students in Western contexts is essential for informing interventions that promote health and wellbeing while encouraging the integration of culturally appropriate and sustainable eating habits in host countries (Millar et al., 2025).

A large body of extant literature has addressed dietary acculturation, influencing factors and health outcomes among international students in the U.S. (e.g., Alakaam, 2016; Alakaam et al., 2015; Alakaam and Willyard, 2020; Almohanna et al., 2015; Wu and Smith, 2016). Although population of Nepalese students in the U.S. continues to grow, few studies conducted among Nepalese students in the U.S. have only focused on academic adjustment (Atteraya, 2021; Vaidya et al., 2024), with limited attention to their dietary transitions. Moreover, food is deeply embedded in festivals and cultural rituals, with seasonal ingredients and specific dishes symbolizing communal values, spiritual beliefs, and ethnic heritage in Nepal (Shakya, 2006; Tamang, 2020). While the traditional Nepalese dish dal bhat tarkari (lentils, rice and vegetables) remains a staple in daily diets, many ethnic foods are consumed less frequently, and most festival-specific dishes are typically reserved for special occasions, rituals, and celebrations, distinguishing them from everyday meals and emphasizing their cultural significance (Gyanwali, 2023). For Nepalese international students, preserving these foodways can play a vital role in maintaining cultural identity and supporting emotional wellbeing during acculturation. Hence, this study aims to explore the continuation of traditional ethnic and festival foods among Nepalese students in the U.S. within a foreign academic and cultural context. Findings will be meaningful in informing the development of culturally responsive support systems that promote both wellbeing and cultural sustainability among Nepalese and international students with similar practices. For Original Research Articles, Clinical Trial Articles, and Technology Reports the introduction should be succinct, with no subheadings. For Case Reports the Introduction should include symptoms at presentation, physical exams and lab results.

## 2 Literature review

### 2.1 Ethnicities, festivals and foods in Nepal

Nepal is home to over 142 ethnic groups speaking 124 languages and following different religions, who observe a wide range of rituals, festivals, and cultural celebrations throughout the year (Thapa-Oli and Yang, 2024). Among these ethnic groups, Chhetri and Brahmin—primarily of Indo-Aryan ancestry originating from India—together make up about 28% of the population (National Statistics Office, 2025a). Approximately 18% are of Mongolian descent, including groups such as Magar, Tamang, Gurung, Rai, Limbu, Sherpa, Bhote, etc., who trace their origins to Mongolia and Tibet (National Statistics Office, 2025a). The Tharu and the Newar account for around 6 and 5% while the remaining 43% consist of various other castes and ethnicities (National Statistics Office, 2025a). Nepal's ethnic diversity also corresponds closely with its geographical zones: people in the high mountain regions are predominantly of Mongolian origin, with linguistic and cultural ties to Tibet; the southern plains are culturally and ethnically similar to northern India and dominated by Terai communities (Madhesis); and the mid-hill regions are mainly inhabited by Brahmin, Chhetri or traditionally dominant castes (Tamang, 2020; Thapa-Oli and Yang, 2024).

The majority of Nepal's population adheres to Hinduism (81.18%), followed by Buddhism (8.2%), Islam (5.08%), Kirat (3.16%), Christianity (1.75%), and other religions (approx. 0.59%) (National Statistics Office, 2025a). Hindus refrain from consuming beef, as cow is considered a sacred animal, and Muslims refrain from consuming pork as it is prohibited by their dietary laws (Tamang, 2016). Likewise, orthodox Brahmin and Chhetri families abstain from consuming pork and buffalo meat due to sociocultural norms (Morimoto, 2020). These families also sacrifice goats during religious rituals and consume goat meat, which, along with chicken and fish, is common across various ethnic groups (Tamang, 2016). Newar communities prefer delicacies made from buffalo meat, while ethnic groups of Tibeto-Burman origin consume yak and sheep meat as well (Gyanwali, 2023; Morimoto, 2020; Tamang, 2016). Some of the ethnic foods of Nepal rooted in ethnic traditions are listed in Table 1. Although many of these foods have roots in specific ethnic traditions, they are now widely shared and enjoyed across diverse ethnic communities throughout Nepal (Gyanwali, 2023; Tamang, 2016). These ethnic foods also reflect the connection between ethnic communities, their geographical regions, and the agricultural practices specific to those areas (Tamang, 2020).

**Table 1:** Some of the ethnic foods rooted in ethnic communities of Nepal.

Ethnic groups	Foods (ingredients and/or preparation)
Indo-Aryan (Brahmin, Chhetri, etc.), Thakuri, Rana	• *Kheer* (sweet dish made of milk and rice) • *Puri* (deep fried wheat bread) • *Alu-ko-achaar* (spicy potato salad) • *Anarsa* (sweet bread made of rice flour, ghee, sugar and white sesame) • *Malpuwa* (sweet syrup-dipped pancake made of wheat flour)
Tibeto-Burman (Magar, Tamang, Rai, Limbu, Gurung, Sherpa, etc.)	• *Dhindo* (thick mush made of millet or corn flour) • *Gundruk* (fermented dried sour food prepared from leafy vegetables) • *Sinki (fermented radish)* • *Tama* (fermented bamboo shoots) • *Momo* (dumplings prepared with thin dough wraps filled with stuffing of minced meat, chopped onion and shallots) • *Thukpa* (noodles with soup) • *Chhurpi* (yak cheese) • *Tsampa* (flour milled from roasted barley) • *Shyafaley* (deep fried wheat or rye dough wrapped in minced meat) • Butter tea
Terai communities	• *Bagiya* (steamed dumpling made with rice flour stuffed with molasses, vegetables or sesame flour) • *Sidra* (mixture of taro yam, fish, butter, spices, tomatoes, and gravy) • *Masaura* (fermented sundried balls made of black lentils paste and vegetables like taro, yam, or colocasia leaf) • *Ghonghi* (water snails) • freshwater crabs and prawns
Newar communities	• *Bara* (pancake made from ground-soaked lentils) • *Yomari* (rice flour with sweet filling of *chaaku* or *khuwa*) • *Chhoila* (buff meat steamed and marinated with rich masala spices and sautéed with mustard oil) • *Chatamari* (Newari pizza with base of rice flour paste with toppings of meat, eggs or beans) • *Dhau* (sweet yogurt) • *Lapsi pau* (sweet and spicy hog plum candy) • *Lakhamari* (sweet dish made of flour, sugar and butter fried in oil)
Traditional (common among Nepalese people)	• *Khalpi* (fermented pickle of ripe cucumber, spices and salt) • *Jeri/jalebi* (syrup-filled confection that is deep fried in oil after fermenting the wheat flour dough) • *Fulaura* (fried black lentil paste mixed with spices) • *Pakauda* (deep fried vegetables and spices mix dipped in gram flour) • *Dahi* (yogurt), *mahi* (buttermilk), *ghiu* (ghee) • *Khuwa* (sweet solid product obtained after boiling milk with sugar) • *Makai- bhatmas* (roasted maize and soyabean) • *Satoo* (flour of roasted maize, soyabean, wheat, etc.) • Fermented pickles of radish, hog plum, *akabare* chili with seasoning

Source: Compiled from Dahal et al. (2005) and (Tamang 2016, 2020).

In addition, cultural and religious celebrations prominently feature communal eating in Nepal, where food serves as a focal point of the festivities (Sapkota et al., 2017). Festivals are mainly observed according to the lunisolar calendar, Vikram Samvat and lunar calendar, Nepal Samvat consisting of 12 months (Shakya, 2006; Tamang, 2016). The new year of Vikram Samvat is celebrated in the month of Baisakh (mid-April) whereas it is celebrated in the month of Mangsir (November-December) according to Nepal Samvat (Shrestha, 2015). While some festivals are celebrated nationwide, others are specific to particular ethnic groups, reflecting their cultural heritage and traditional foods. The major festivals celebrated in Nepal that highlight foods are presented in Table 2.

**Table 2:** Some of the major festivals and festival foods of Nepal.

Festivals (time of celebration)	Foods (ingredients and/or preparation)	Ethnic groups
Dashain (September-October)	• Various dishes made of goat meat • Other homemade delicacies	All
Tihar (October/November)	• *Selroti* (ring-shaped fried fare prepared by mixing rice flour paste, sugar, ghee and spices) • *Bhai masala* (dry fruits, citrus fruits and sweets) • *Anarsa* (sweet bread made of rice flour, ghee, sugar and white sesame) • *Finiroti* (deep-fried crispy multilayered bread made of rice flour, ghee, water, salt and sugar) • *Bagiya* (steamed dumpling made with rice flour stuffed with molasses, vegetables or sesame flour)	All
Chhath (October-November)	• *Thekuwa* (deep fried crispy sweet food made of wheat flour, jaggery, ghee, and fennel seeds)	Terai communities
Maghe Sankranti (Mid-January)	• *Chaaku* (prepared from sugarcane jaggery) • *Teel-ko-laddu* (sesame seeds) • *Khichadi* (rice and split mung beans) • Yam, sweet potatoes, etc.	All
Janai Purnima (August-September)	• *Kwanti* (soup curry of nine beans)	All
Yomari Punhi (November/December)	• *Yomari* (rice flour with sweet filling of *chaaku* or *khuwa*)	Newar communities
Tamu, Sonam and Gyalpo Lhosar (various between November-March)	• *Aalum* (buckwheat flour kneaded and developed into the shape of animal of the year and then boiled) • *Baabar* (deep fried thick slurry of rice flour) • *Guthuk* (soup made with nine ingredients including yak's meat, vegetables, beans and cheese) • *Khapsey* (deep fried twisted wheat dough flavored with butter, sugar, salt, milk and condiments) • *Selroti* (ring-shaped fried fare prepared by mixing rice flour paste, sugar, ghee and spices)	Ethnic groups of Tibeto-Burman origin (Gurung, Tamang, Sherpa, etc.)
Teej (August/September)	• *Darr* including *khir*, sweets and other homemade delicacies	All
Asar 15 (June/July)	• Mix of *dahi* (yogurt), *chiura* (beaten rice) and fruits	All
Shrawan 15 (July/August)	• *Khir* (sweet dish made of milk and rice)	All
Jatras (various occasions throughout the year)	• *Samaya baji* (platter comprising of fried boiled egg, beaten rice, bara, curry of bamboo shoots, black eyed beans and potatoes, spinach, spicy soybeans or peanuts, grilled meat curry, etc.) • Other newari ethnic foods	Newar communities

Source: Compiled from Anderson (2024), Gyanwali (2023), Sapkota et al. (2017), Shakya (2006), and Tamang (2016).

Major festivals such as Dashain and Tihar are widely celebrated Hindu festivals, along with Maghe Sankranti and Janai Purnima, though the associated traditions and food customs often vary across ethnic groups (Gyanwali, 2023; Shakya, 2006). Newar communities celebrate distinct festivals like *Yomari Punhi* and various *jatras* (religious processions) that showcase Newari culture and cuisine (Shakya, 2006). Similarly, ethnic communities from Terai observe Chhath and other local festivals that highlight regional and ethnic food practices while groups of Tibeto-Burman descent, such as the Tamang, Gurung, and Sherpa, observe their New Year known as Sonam, Tamu and Gyalpo Lhosar respectively, rooted in Buddhist traditions and marked by traditional festive foods (Tamang, 2016).

While many festivals involve the preparation and sharing of foods, others are marked by complete (abstaining entirely) or partial fasting (eating only certain foods at specific times) (Sapkota et al., 2017). For instance, during Teej, women fast for an entire day for the longevity of their husbands, preceded by a feast known as *darr*, which includes a variety of dishes (Tamang, 2016). Fasting is also observed by Hindus during Saraswati Puja, Shivaratri, Chhath, Ekadashi (twice a month), and weekly fasts, and Muslims during Ramadan, reflecting religious devotion and vows (Gyanwali, 2023; Kalra et al., 2015). The interplay between ethnic diversity, seasonal festivals, and traditional food practices reflects the cultural richness and complexity of Nepal's sociocultural landscape.

### 2.2 Dietary acculturation of international students and influencing factors

Dietary acculturation pertains to how individuals from one cultural background modify their food-related behaviors, preferences, and consumption patterns when they migrate to a new sociocultural environment (Satia-Abouta, 2010; Satia-Abouta et al., 2001). This process is marked by both change and continuity as migrants navigate their relationship with traditional foods in light of new influences (Satia-Abouta et al., 2002). It is an evolving, interactive, and multidirectional process, wherein both the immigrant population and the dominant host culture exert mutual influence on dietary behavior (Satia-Abouta, 2003, 2010). (Satia-Abouta et al. 2002) proposed a dietary acculturation model in which demographic, socioeconomic, cultural, and psychological variables interact and function through complex mechanisms that may shape food practices in various ways. Individuals may retain their traditional dietary patterns; adopt the host country's food culture; or find a balance between the two through bicultural dietary adaptation (Satia-Abouta, 2003; Satia-Abouta et al., 2002). Moreover, factors such as sociodemographic, cultural values or religious beliefs may directly shape dietary acculturation, independent of exposure to the host country's food environment, signifying the depth and resilience of culturally embedded food practices (Satia-Abouta, 2003).

For international students, higher education settings provide an inherently multicultural environment that can shape their food-related behaviors profoundly (Millar et al., 2025; Shi et al., 2021). Daily interactions with peers from diverse backgrounds and exposure to new institutional and cultural norms may impact attitudes toward food, beliefs about health, and perceptions of acceptable dietary practices, thereby influencing students' food choices (Alakaam, 2016; Edwards et al., 2010). A variety of social and demographic variables have been identified as significant determinants of dietary acculturation in this population. These include social interaction and networks, living arrangements, financial stability, gender, family support, and length of residence in the host country (Alakaam et al., 2015; Brittin and Obeidat, 2011; Chai et al., 2019; Mensah et al., 2022). For instance, research among international students at universities in the Midwestern and Northeastern U.S. found that those living with spouses reported fewer changes in dietary behavior, while those with children reported more pronounced shifts (Alakaam et al., 2015). This was attributed to children's preferences for non-traditional foods and the limited time available for preparing culturally familiar meals. Additionally, peer influence, especially in the context of dining out or participating in communal food preparation and sharing, was found to hinder and facilitate the continuity of traditional food practices respectively, as reported among the Black, Asian and minority ethnic students in the U.K. (Mensah et al., 2022).

Studies have reported that cultural variables such as ethnicity, religion, and traditional values influence not only food choices but also the meanings attached to food and eating practices (Alakaam et al., 2015; Brittin and Obeidat, 2011; O'Sullivan and Amirabdollahian, 2016; Wright et al., 2021; Yan and FitzPatrick, 2016). Yan and FitzPatrick (2016) emphasized that students from culturally proximal regions, such as Latin America, tended to face fewer dietary adaptation challenges compared to students from culturally distinct regions like East and South Asia, who reported more concerning barriers in adapting to Western food systems. Brittin and Obeidat (2011) documented how religious dietary laws led to the avoidance of pork and alcohol, both of which were among the most rejected items by international Muslim students. Likewise, Wright et al. (2021) found that food was a critical link to identity, familial heritage, and religious values for international students. When access to cultural foods such as halal or region-specific ingredients was limited, students were compelled to make dietary adjustments that did not align with their cultural traditions or beliefs, resulting in a dissonant acculturation experience.

Psychological and individual-level factors, including perceptions of food quality and healthiness, emotional states, food-related memories, time availability, cooking skills, and attitudes toward traditional vs. Western food also shape dietary acculturation among international students (Alakaam et al., 2015; Brown et al., 2010; Hartwell et al., 2011; Lee et al., 2020; Mensah et al., 2022; O'Sullivan and Amirabdollahian, 2016; Wu and Smith, 2016; Yan and FitzPatrick, 2016). Time constraints and lack of cooking skills were observed to be especially impactful, as many students reported skipping meals or abandoning traditional food preparations due to their academic workload and lack of culinary skills (Lee et al., 2021; Mensah et al., 2022). Whereas Yan and FitzPatrick (2016) found that international students adopted multiple approaches such as seeking ethnic grocery stores, ordering from restaurants offering traditional cuisine, or moving off-campus to have greater autonomy over cooking facilities to fulfill their taste preferences. Findings from a study conducted by Wright et al. (2021) highlighted that the sensory memories associated with food from home were powerful emotional anchors for international students in the U.S., affecting their food choices and psychological wellbeing.

Environmental and structural factors, including food cost, availability, accessibility, campus food infrastructure, and the broader food retail environment, also intersect with and influence dietary acculturation trajectories (Brown et al., 2010; Dean et al., 2024; Hartwell et al., 2011; Lee et al., 2020; Wu and Smith, 2016). Limited access to traditional food items, lack of culturally familiar dining options, and high costs were cited as major constraints that forced international students to adapt to mainstream dietary patterns that may not align with their preferences (Dean et al., 2024). Such external pressures were found to accelerate the process of dietary acculturation, leading to nutritional compromises or cultural disconnection.

## 3 Purpose and research questions

The purpose of this study was to explore the continuation of traditional food practices, particularly ethnic and festival foods, by Nepalese students in the U.S., the influencing factors, and significance attached to the maintenance of these foods and cultural traditions in a new environment.

To guide this exploration, the study addressed the following research questions:

RQ1: What ethnic and festival foods and practices do Nepalese students continue while living in the U.S.?
RQ2: What factors influence Nepalese students' ability to sustain ethnic and festival food traditions in the U.S.?
RQ3: How do Nepalese students interpret the significance of sustaining ethnic and festival food traditions in the U.S.?

## 4 Materials and methods

Qualitative method, with its virtue in examining contextual phenomena that are difficult to quantify and offering rich insights, was used for this study (Creswell and Poth, 2016). A phenomenological approach was adopted to capture the shared meaning of the continuation of ethnic and festival food practices among Nepalese students in U.S. universities (Padilla-Díaz, 2015). Due to the scarce research on the continuation of traditional foods and its implications among international Nepalese students in the U.S., a phenomenological approach was well-suited to deeply explore and interpret their lived experiences. The study was approved by the Institutional Review Board at Iowa State University (IRB ID 24-511, 12/18/2024).

### 4.1 Sampling

Purposive sampling was employed to recruit participants who were best positioned to provide rich, relevant data (Tisdell et al., 2025). The population of this study is the Nepalese students enrolled in universities across the Midwestern U.S. The Midwest was selected as the research site due to its significant representation of Nepalese students, with Nepal ranking among the top five countries of origin for international students in six states of the region: Kansas (5.3%), Minnesota (6%), Missouri (4.1%), Nebraska (5.3%), South Dakota (22.4%), and Ohio (5.9%) (Institute of International Education, 2025b).

Participants between 18 and 35 years old were recruited for the study. This group was chosen because it aligns with the predominant age group enrolled in higher education institutions in both Nepal and the U.S. (National Statistics Office, 2025b; U. S. Department of Education, 2023). Only students who had resided in the U.S. for more than 3 months were included to exclude those in the early, honeymoon phase of cultural transition, often characterized by enthusiasm and optimism (Fitzpatrick, 2017). Additionally, only participants who had not completed high school or earlier education in the U.S., were recruited since it was assumed that early acquaintance with American culture could have possibly limited exposure to Nepalese ethnic and festival foods and traditions.

### 4.2 Data collection

A semi-structured interview format was adopted, enabling flexibility for follow-up questions, clarification, and the exploration of emerging themes during both focus groups and interviews (Glesne, 2016). Moderator and interviewer guides were developed to examine various dimensions, including cultural food beliefs, values, and memories, continuation of ethnic and festival foods and practices, and factors influencing the maintenance of these foods and practices. These questions were guided by literature review on international students' dietary acculturation and traditional Nepalese ethnic and festival food culture. To elicit participants' emotional and sensory memories associated with Nepalese ethnic and festival foods, visual stimuli in the form of food images were presented during the interviews and focus groups. The guide was reviewed by a qualitative research specialist to ensure question clarity and reliability. It was then pilot tested with two Nepalese students to assess content validity, whose feedback led to minor revisions aimed at improving the wording and minimizing possible confusion. Data collection involved both focus group discussions and individual interviews, conducted in English language. While focus groups enabled the exploration of shared cultural narratives and collective perspectives, individual interviews offered deeper insight into personal experiences (Michell, 1999).

#### 4.2.1 Focus groups

In-person focus groups were conducted with Nepalese students enrolled at Iowa State University (ISU), chosen for their accessibility to the research team. Email addresses were obtained via the ISU Office of the Registrar, and all the Nepalese students received an invitation that included a study overview, a demographic survey, and an informed consent form. Of the 21 students who initially responded, three withdrew due to scheduling conflicts. Ultimately, three focus groups were held with seven, six, and five participants, with a total of 18 focus group participants. The focus group sessions were conducted in February 2025. Each session lasted for an average of 95 min and was facilitated by the lead researcher, with two team members taking notes. Written consents were obtained prior to the beginning of the focus group sessions. Each participant received a $40 gift card in appreciation of their time.

#### 4.2.2 Interviews

Individual interviews were conducted with Nepalese students from various Midwestern universities. The interviews were conducted online due to practical constraints related to time and financial resources required for travel across the Midwestern United States. This approach also aligned well with the study sample, as university students are generally familiar and comfortable with virtual communication technologies, minimizing any potential impact on the depth or quality of participant responses. The primary researcher approached Nepalese Student Associations of the universities via Facebook, Instagram and personal network, who shared digital flyers for recruitment. The flyers included a study description, demographic survey, and consent form. Of 48 survey respondents, two were excluded for not meeting the eligibility criteria. From the remaining pool, 13 students were initially invited. To ensure diversity in terms of ethnicity, religion, academic and institutional backgrounds, 10 more individuals were later contacted, with five of them participating in the interview, resulting in a total of 18 completed interviews. Signed digital consent was obtained prior the interviews which were conducted via Webex between February and March 2025 and lasted for an average of 62 min. Each participant received a $40 gift card. Data saturation was deemed achieved with 18 interviews when no new themes emerged.

To enhance credibility, note-takers documented responses and conducted member-checking at the end of each session. The lead researcher transcribed all focus group and interview recordings using Otter.ai, reviewed the transcripts for accuracy and de-identified them.

#### 4.2.3 Demographic characteristics of participants

The study involved 36 Nepalese students, enrolled in universities across the Midwestern U.S. of these, 18 participated in focus group discussions, while the remaining 18 completed online interviews. The participants had a mean age of 26.19 years (*SD* = 3.77). The sample included 20 male and 16 female students from diverse ethnic backgrounds, including Brahmin (*n* = 20), Newar (*n* = 8), Chhetri (*n* = 4), Madhesi (*n* = 2), Tamang (*n* = 1), and Kami (*n* = 1). Most participants identified as Hindu (*n* = 34), with two identifying as Buddhist. In terms of academic level, the group comprised undergraduate (*n* = 12), master's (*n* = 11), and doctoral (*n* = 13) students. The duration of their residence in the U.S. ranged from 6 months to 7 years. Table 3 provides a detailed breakdown of participants' demographic characteristics.

**Table 3:** Demographic characteristics of participants.

Characteristics	Number of participants	
	Focus groups (*n* = 18)	Interviews (*n* = 18)
Age in years (Mean ± SD)	26.19 ± 3.77	
Generation		
Millennials (born 1981–1996)	6	5
Generation Z (born 1997–2012)	12	13
Sex		
Male	11	9
Female	7	9
Ethnicity		
Brahmin	11	9
Newar	4	4
Chhetri	1	3
Madhesi	1	1
Tamang	0	1
Bishwokarma	1	0
Religion		
Hinduism	18	16
Buddhism	0	2
Academic level		
Graduate (Doctoral degree)	7	6
Graduate (Master's degree)	5	6
Undergraduate	6	6
University		
Iowa State University	18	0
University of Nebraska-Lincoln	0	2
North Dakota State University	0	1
University of Kansas	0	1
Kansas State University	0	1
University of South Dakota	0	3
University of Illinois at Urbana- Champaign	0	4
Southern Illinois University-Carbondale	0	1
Minnesota State University-Mankato	0	2
University of Wisconsin-Madison	0	1
Missouri State University	0	1
University of Missouri-Columbia	0	1
**Length of stay in the U.S**.		
More than 5 years	2	2
3–5 years	2	3
2–3 years	3	3
1–2 years	6	6
6 months to 1 year	5	4

### 4.3 Data analysis

Thematic analysis was conducted to interpret recurring patterns and generate meaningful insights, rather than merely cataloging surface-level trends (Clarke and Braun, 2017). This iterative process involved comparing codes, organizing them into broader categories, and identifying emergent themes. A codebook was developed through initial coding carried out by the primary researcher using MAXQDA Analytics Pro 24. The analysis was conducted using inductive coding strategy to allow new themes to emerge directly from the data without being constrained by pre-existing theories (Glaser and Strauss, 1967). This approach allowed themes to emerge directly from participants' narratives, capturing novel insights, culturally specific patterns, and context-dependent nuances unique to Nepalese students' experiences. To maintain analytical rigor, the researcher held regular peer debriefing sessions with another research team member with extensive experience in qualitative research, which facilitated refinement of a preliminary codebook through collaborative feedback. To strengthen the reliability of the findings and reduce researcher bias, a trained external coder independently analyzed a subset of the data using the same coding framework. The codebook was further refined through peer discussions, repeated review of transcripts, and consideration of alternative interpretations. The final coding resulted in the synthesis of key concepts into overarching themes that captured the core insights of the study.

In alignment with the criteria of trustworthiness established by Lincoln and Guba (1985), multiple strategies were employed to ensure credibility, transferability, dependability, and confirmability. Credibility was enhanced through pilot testing of interview protocols, member-checking with participants, and triangulation of methods via both focus groups and individual interviews. To support transferability, purposive sampling ensured demographic and academic diversity among participants, while data saturation reinforced the applicability of findings to comparable populations. Dependability was maintained through a comprehensive audit trail documenting each phase of the research process from instrument development and data collection (e.g., audio recordings, transcripts, and field notes) to data analysis and synthesis (Tisdell et al., 2025). Confirmability was established through detailed documentation and researcher triangulation, with multiple researchers independently coding and analyzing data, enabling independent verification of findings and minimizing subjective bias in interpretation.

### 4.4 Reflexivity statements

As qualitative research positions the researchers themselves as the key instrument, it is significant to disclose authors' positionality, which may have influenced approach to this study and the interpretation of the findings (Creswell and Poth, 2016).

The first author, who served as the primary researcher and led the study design, is a Ph.D. student in agricultural education and communication with training and coursework in qualitative research methodologies. Originally from Nepal and currently pursuing graduate studies in the U.S., she shares lived experiences with the study participants. This positionality as an insider offered valuable cultural insight that enriched data interpretation.

The second author, an associate professor in agricultural communication, brings personal experience as an international student from China, having completed her master's and doctorate degrees in the U.S. Drawing on her background and expertise in qualitative research, she provided guidance on the organization and expression of themes throughout the analysis.

The third author is an assistant professor in science communication whose research focuses on exploring the use of communication as a tool for amplifying voices of the marginalized. She has over 10 years' experience working with communities from diverse backgrounds. Her contribution and involvement in the study was grounded on her experiences and research focus.

The fourth author is a professor of journalism with a specialization in political communication and cross-cultural media studies. Having been an international student herself, she was able to relate to the topic and draw on her personal experiences. Her research expertise regarding the role of interpersonal communication and media messaging about diets contributed to the study.

The external coder in this study is a Ph.D. student in agricultural education at Iowa State University. Originally from India, she moved to the U.S. to pursue her doctoral studies. She has received advance coursework in qualitative inquiry and actively participated in various qualitative research projects. Hence, she brings relevant training and practical experience in qualitative research for qualitative coding of the data for this study.

## 5 Results

Participants' responses revealed six central themes related to the continuation of their ethnic and festival food practices while living in the U.S. Participants were identified with unique number followed by alphabet ‘S'. S1 to S18 represent participants from focus groups while S19 to S36 represent participants from interviews. Each participant's quote includes a notation indicating the student's alphanumerical identity, gender and academic level, with F = female, M = male, G = graduate student, and UG = undergraduate student.

### 5.1 RQ1: Ethnic and festival foods and practices continued in the U.S.

Two key themes emerged from participants' responses regarding ethnic and festival foods and practices continued in the U.S.: 1) ethnic and festival foods on the table, and 2) selective continuation of fasting food practices.

#### 5.1.1 Ethnic and festival foods on the table

Along with the continuation of traditional Nepali staple meal *dal bhat tarkari* (pulses, rice and vegetables) as a part of their regular dietary routine in the U.S., many respondents described efforts to maintain the preparation and consumption of specific ethnic and festival foods tied to their cultural and religious heritage. Festival-specific foods commonly mentioned included mutton during Dashain, *selroti* during Tihar, *khichadi, ghiu chaaku, laddu* during Maghe Sankranti, and *kheer* for other festivals and celebratory occasions. While some participants were able to prepare these traditional dishes on their own during festivals, others recalled being invited by fellow Nepalese peers to have such festival foods. For instance, one student noted: “We do have a Nepalese family who is pretty close to us. They invited us for Maghe Sankranti, where we had most of the homemade [festival food] items of chaaku, laddu and other stuff.” (S34, M, G)

In addition to festival-specific foods, participants reported continuing a variety of ethnic Nepali dishes such as *momo, pakauda, gundruk, tama, thukpa, roti* of wheat, millet and buckwheat and various fermented pickles. One participant highlighted the persistence of this food culture: “We try to make momo whenever we can, which I think is a continuation of what we did back in Nepal” (S15, M, UG). Another participant shared preparing curry and pickle from traditional fermented food gundruk that she “continued as a cultural food from Nepal” (S22, F, UG). Only a few participants from specific ethnic communities shared experiences of maintaining ethnicity-based foods. For instance, a few students from Newar background mentioned preparing delicacies such as *bara, chatamari, yomari*, and Madhesi background mentioned preparing *bagiya* during special cultural occasions. A participant from the Madhesi community stated: “During festival seasons, we have typical meals. So, in Mangsir, the last month, we made bagiya, that's a typical Terai food” (S5, M, G). Thus, although not as frequent or identical to the practices back home, participants reported continuing to celebrate festivals and occasions by preparing and sharing ethnic and festival foods whenever feasible.

#### 5.1.2 Selective continuation of religious fasting practices

In addition to maintaining certain food practices rooted in cultural and religious identity, such as avoiding beef due to Hindu beliefs and abstaining from pork in accordance with specific ethnic traditions, a few participants also reported continuing religious fasting practices and dietary restrictions during specific holy days and festivals such as Shivaratri, Saraswati Puja, and Ekadashi. One participant shared: “I didn't use to eat meat in Ekadashi back in Nepal. So, if I know it's Ekadashi here, I don't eat meat... I do fast on Tuesdays. And I've been continuing that from Nepal.” (S30, M, G)

However, several participants acknowledged challenges in maintaining consistent observance of these religious food restrictions, largely due to the mismatch between the Nepali lunar calendar and the Gregorian calendar used in the U.S. As a result, many students admitted to unintentionally overlooking such days unless reminded by family members. For instance, one student stated: “Yesterday, it was Shivaratri. … And I think my roommate's parents also told her it's better to avoid meat for the day. So, we avoided meat (S35, F, UG).” Others reported selectively maintaining some fasting traditions while letting go of others due to practical constraints such as academic schedules and energy demands. A participant reflected: “I fast only during Shivaratri. … I have my own work for the college, and it's difficult to fast the whole day, so I do not follow fasting procedures like I did in Nepal (S20, F, G).” These findings portray how Nepalese international students adapt fasting practices to fit the rhythms of academic life and the realities of living abroad. Selective continuation, external reminders, and negotiated adaptations reflect how they balance cultural preservation with situational pragmatism.

### 5.2 RQ2: Factors influencing students' ability to sustain ethnic and festival food traditions

Two central themes were identified as factors influencing Nepalese students' ability to sustain ethnic and festival food traditions in the U.S.: 1) communal celebration of ethnic and festival foods, and 2) barriers to continuing ethnic and festival foods.

#### 5.2.1 Communal celebration of ethnic and festival foods

A prominent theme that emerged across participant narratives was the collective nature of continuing ethnic and festival food practices. Rather than preparing these foods individually, most participants emphasized celebrating and cooking in groups, often with friends, apartment-mates, or members of the broader Nepalese community and students' organization in the university. This communal aspect appeared central to maintaining culinary traditions and cultural identity. Numerous participants highlighted the shared preparation of momo as a common activity during weekends and academic breaks. One student remarked: “Whenever we are making momo … or something that represents Nepali food, we normally invite a few of the Nepalese people around, and then we do a small gathering and have it and enjoy.” (S35, F, UG)

Beyond informal gatherings, institutional and community-based efforts also played a central role in preserving festival food practices. Many participants attributed their ability to engage with traditional festival foods to the initiatives led by Nepalese student organizations or local community groups. One participant shared: “I do not cook festival foods … but I get the opportunity to have them because there is a Nepalese community in our university. So, during the festivals, they conduct some gatherings, and then they cook such food” (S36, M, G). Others reflected on how these communal efforts supported the continuation of food practices specific to their ethnic identity. A participant from the Newar community described celebrating Yomari Purnima with joy:

I come from a Newar community, so we have a festival every month in Nepal. And we can't celebrate all the festivals here [in the U.S.], but we try to celebrate some of the festivals that we can. One good example is we make yomari in Yomari Purnima … If you are alone, you may not be able to do it. But we have a Nepalese community, and there are a couple of people with Newari backgrounds. So, all of us from the whole community gather and make yomari. Not only did we continue it, but we could also promote and celebrate [it] together, so I feel happy and good (S31, M, G).

Participants repeatedly emphasized how such collective practices strengthened their sense of belonging, cultural identity, and cultural wellbeing. These food-centered gatherings became symbolic of cultural continuity and emotional support in a foreign environment. A student explained how “gatherings while making momo” served as a medium to “meet with the Nepalese students,” “create a sense of community” far from home and connect them to their culture (S35, F, UG). Another participant emphasized the unifying role of food in diaspora life: “Food is the only way that we can connect with each other here as Nepalese. So, the main goal of cooking all these foods and gatherings is actually being together” (S22, F, UG). These findings underscore the importance of social networks and collective cultural practices in sustaining traditional foodways and fostering a sense of community among Nepalese students in the U.S.

#### 5.2.2 Barriers to continuing ethnic and festival foods

Despite strong cultural ties to traditional food practices, many participants identified a range of barriers that hindered the continuation of Nepalese ethnic and festival foods in the U.S. Time emerged as one of the most frequently cited obstacles. Participants noted that academic responsibilities and busy schedules often interfered with their ability to prepare or celebrate with traditional foods during major festivals. One participant highlighted the difficulty of coordinating such efforts within student life: “I don't think we have time to follow everything [during festivals]. We try to make festival foods during Dashain and Tihar in a group of people,… but then again, it depends upon everyone's schedule” (S19, M, G). Another student expressed how the academic calendar often overlapped with Nepalese festivals, making celebration nearly impossible: “I wish [I could prepare festival foods], but … whenever it's our festive season, it's peak period of our semester. So, it's been 4 years since I'm here, and I have never gotten a chance to actually celebrate the festivals.” (S29, F, G)

Lack of experience with preparing traditional foods also posed a significant challenge for some participants. One student shared that her inability to cook prevented her from preparing “selroti, arsa, chini roti” that used to be “cooked during the festivals” back in Nepal. She added “If I had known how to cook them, I would have definitely made them” (S7, F, G). Another participant shared a personal anecdote about an unsuccessful attempt to prepare an ethnic Nepalese dish: “I love dhindo a lot, but I don't know how to cook. Me and my husband tried to prepare dhindo... One time, it was too hard, ... another time it was slurry.” (S26, F, G)

Another prominent barrier reported by participants was the lack of access to specific foods and ingredients necessary for preparing traditional Nepalese dishes. The absence of culturally essential meats and other ingredients was particularly challenging for students from specific ethnic communities. For instance, one student shared that coming from a Newar community, she “consumed buff on every occasion, festival, celebration and weekends” back in Nepal, but she has “never been able to find it in the past 6 years in the U.S.” (S33, F, G). Another participant reflected on the broader difficulty of sourcing multiple components needed for traditional festival meals: “I cannot source everything that we used to prepare back in Nepal [during the festivals] here in the U.S.” (S32, F, UG)

In addition, one notable barrier involved concerns over bringing ethnic foods, particularly fermented items, from Nepal due to U.S. customs and airport security regulations. Several participants shared apprehensions about potential misunderstandings or penalties. One student recounted a story that discouraged her from transporting traditional fermented vegetables:

I wanted to bring some of the fermented foods like gundruk, but I was so scared that they would not allow me when I come here, so I didn't. Because … when one of my friends came to the U.S. for the first time, she brought some gundruk with her, and they thought it was marijuana. So, she was at the airport for like 12 h … and our international advisor had to get involved in the situation.” (S33, F, G)

Another participant mentioned not bringing fermented pickle “because it's liquid, so I am never sure if the customs will allow me to bring it” (S19, M, G). These findings illustrate how logistical, practical, and structural factors intersect to limit the full continuation of Nepalese ethnic food traditions among the international students. While the intent to maintain cultural food practices remains strong, such barriers considerably affect students' ability to recreate the full scope of traditional Nepalese culinary experiences in the U.S.

### 5.3 RQ3: Interpretation of significance of sustaining ethnic and festival food traditions

Participants interpreted the significance of sustaining ethnic and festival food traditions in ways that coalesced around two themes: 1) memories related to ethnic and festival foods, and 2) emotional resonance through ethnic and festival foods.

#### 5.3.1 Memories related to ethnic and festival foods

The digital visual prompts of ethnic and festival foods served as effective catalysts, encouraging participants to reflect on their past experiences with cultural foods and the significance these foods held in their familial and social lives in Nepal. Several participants described deep emotional associations between traditional foods and family gatherings. One participant reflected on how momo served as a “way for my family to get together because me, my cousins and my parents used to get together to make momo most of the time. So, it's a lot of memories of us being together” (S15, M, UG). Festival-specific dishes such as mutton prepared during Dashain were also strongly associated with extended family traditions, hospitality, and celebration. As one student fondly recalled: “During Dashain, we would buy a whole goat and cook everything. It would be enjoyed for the whole half month long festival. Whenever we had relatives, we would cook the meat in various different ways.” (S25, M, UG)

Beyond family traditions, participants also recalled experiences of ethnicity-based foods sharing across Nepal's diverse ethnic landscape. One participant described how the food practices of his Terai heritage extended into act of cultural exchange among peers: “I belong to Terai community, and we used to make khajuri in the festival of Chhath. And after the Chhath would be over, we used to bring tons of khajuris to Kathmandu and share with our friends” (S23, M, G). Another participant recounted his experience of engaging with Newari cuisine through friendships and local festivals, illustrating how cultural foods often transcend household boundaries: “Since most of our closest friends were Newar, we used to be invited a lot into jatras and festivals where I could enjoy Newari foods.” (S34, M, G)

These narratives illustrate how ethnic and festival foods function as repositories of memory, community, and identity among Nepalese students in the U.S. They not only represent nourishment but also embody shared cultural rituals, intergenerational bonds, and cross-cultural exchanges that continue to hold significance for the students.

#### 5.3.2 Emotional resonance through ethnic and festival foods

Participants revealed that the continuation or disruption of ethnic and festival food practices in the U.S. held deep emotional significance, often linked to longing, comfort, and a sense of cultural loss or continuity. Food emerged not only as a material practice but as an emotionally charged symbol of home, family, and cultural identity. For many participants, the inability to replicate certain traditional foods evoked strong feelings of longing. One participant expressed this vividly in relation to ethnic Nepalese fermented pickles: “Those pickles, I just miss those because I can't make those at all even if I get the ingredients because we don't have a lot of sunlight over here to make those” (S22, F, UG). Other participants reflected on the irreplaceable nature of food prepared by loved ones, particularly in the context of festivals. A student shared his yearning for the taste of his mother's cooking during Dashain: “One of the most important things I miss a lot is my mother cooking meat for me. She used to cook extremely delicious meat, and now I'm craving a lot for that (S30, M, G).”

In some cases, participants actively sought comfort through ethnic Nepalese foods as a coping mechanism for homesickness and emotional distress. A participant shared how these foods offered a sense of emotional grounding:

Whenever we feel down, we kind of hang out and have typical Nepalese foods for comfort. And we all miss our family; we all miss our food. But having such foods together reminds us that we are not alone … and every time we eat Nepalese food, it reminds me of my home (S27, F, UG).

Beyond immediate emotional responses, participants also expressed feeling of cultural guilt and anxiety over the discontinuation of cultural food traditions: “I think what scares me is, what are we going to give our children in the future because now we are starting to forget all our food traditions that used to be carried from our family” (S19, M, G). Collectively, these narratives underscore how ethnic, and festival foods are embedded with emotional meaning and play a critical role in maintaining psychological wellbeing among Nepalese students in the U.S.

## 6 Discussion

This study intended to explore the continuation of ethnic and festival foods and practices among Nepalese students studying in the U.S. along with factors influencing the continuation and significance attached to it. The findings revealed that despite physical and contextual dislocation, Nepalese international students in the U.S. demonstrated agency and intention in preserving their traditional foodways through the continuation of ethnic and festival foods. These culinary practices served not only as a form of sustenance but as an active means of cultural preservation, closely tied to identity, heritage, and cultural and emotional wellbeing as suggested by Wright et al. (2021). Although they were not able to celebrate all the festivals and relish festival foods as they did back in Nepal, continued consumption of some festival and ethnic delicacies illustrated a determined effort to retain traditional food practices even while adapting to a new food environment. Additionally, the study uncovered a pattern of selective religious food practice continuity, with students continuing certain fasting rituals like those observed on Shivaratri or Ekadashi while abandoning others due to contextual limitations. This selective continuation suggests a negotiated form of acculturation. Students actively engaged in strategic cultural retention, choosing which traditions to maintain and which to adjust based on relevance, feasibility, and emotional significance.

One of the most salient findings was the communal nature of ethnic food practices. Cooking and eating together in groups, was a profound expression of social bonding and collective cultural identity. These gatherings were often facilitated by friends, apartment groups, Nepalese student organizations, or broader diasporic communities, helping mitigate isolation during festivals and special occasions and strengthening a sense of belonging. Such collective practices enabled students to engage more fully with their traditions and to share them with peers from diverse backgrounds, promoting interpersonal solidarity and emotional wellbeing. This aligned with previous literature (Alakaam et al., 2015; Yan and FitzPatrick, 2016), which recognizes that immigrant food practices are often upheld through communal reinforcement and shared values, especially in diasporic contexts. Moreover, these results support the idea that cultural food practices do not necessarily disappear post-migration but are re-contextualized and collaboratively negotiated, often taking hybrid or adaptive forms within social groups (Satia-Abouta, 2010).

Despite strong cultural commitments, the study also underscored multiple barriers that hindered the full continuation of traditional Nepalese foodways. Chief among these were time constraints, especially due to academic workloads and misalignment between U.S. academic calendars and Nepalese festivals. Additionally, limited cooking skills, lack of access to authentic ingredients, and regulatory concerns around bringing fermented items from Nepal created significant logistical hurdles. These findings echo a substantial body of literature that has identified time constraints and limited cooking skills (Brittin and Obeidat, 2011; Lee et al., 2021; Mensah et al., 2022), and the unavailability of traditional food items (Alakaam et al., 2015; Dean et al., 2024; Wu and Smith, 2016) as major barriers faced by international students in maintaining their traditional food practices. These findings also reinforce dietary acculturation model by highlighting how environmental barriers such as food availability and customs regulations can impede cultural food maintenance (Satia-Abouta et al., 2002).

Participants' narratives revealed how ethnic and festival foods function as emotional anchors, serving as symbols of love, memory, and home. Memories of family meals, specific dishes prepared by parents and the sensory stimuli associated with Nepalese food traditions elicited feelings of nostalgia, longing, and comfort. This emotional resonance affirms prior studies that position food as a cultural archive, a medium through which diasporic individuals re-experience and reaffirm their identities and familial connections (Hartwell et al., 2011; Wright et al., 2021). The emotional value of these foods also appeared to play a coping role in students' lives, helping alleviate homesickness and fostering a sense of emotional wellbeing which is consistent with findings from a review conducted by Millar et al. (2025). This calls for efforts to facilitate access to cultural food ingredients, provide communal cooking spaces, and recognize the emotional and cultural needs tied to food could enhance international students' sense of belonging and wellbeing. At the same time, feelings of cultural loss and guilt emerged when students expressed concerns about forgetting traditional food practices or not being able to pass them on to future generations. This reveals the intergenerational dimension of cultural food transmission, where food is not only a personal choice but a moral and cultural responsibility.

Overall, the findings of the study support the broader understanding that continuation of traditional food practices during the course of dietary acculturation in a foreign environment, is a dynamic, multidimensional process, shaped by cultural, psychological, social, and environmental factors (Satia-Abouta, 2003). However, the findings extend existing dietary acculturation frameworks by demonstrating that food-related adaptation among international students involves not only changes in dietary intake but also the continuation of culturally meaningful foodways, including festival foods, communal food practices, and religious food traditions. The study further refines current models by highlighting the role of social networks and community organizations in sustaining cultural food practices and by illustrating how cultural continuity is actively negotiated in response to environmental, social, and institutional constraints. Rather than representing a static outcome, the retention of traditional foodways emerged as a dynamic process through which students maintained cultural identity, belonging, and emotional wellbeing while adapting to life in a new cultural context.

### 6.1 Limitations

This study has some limitations. First, although we made efforts to recruit Nepalese students from varied ethnic backgrounds, the sample did not fully capture the ethnic and religious diversity of the broader Nepalese population, limiting generalizability. Second, conducting the study in English, rather than participants' first language, Nepali, may also have constrained the depth and clarity of responses. Finally, as a qualitative study with a small sample size, the findings are exploratory and context specific. Future research employing larger samples and diverse methodological approaches, including quantitative or mixed methods, is needed to validate and extend these insights into international students' dietary acculturation.

## 7 Conclusions and recommendations

Unlike existing dietary acculturation research, which predominantly emphasizes dietary changes and host-country food adoption, this study examines how international students actively sustain ethnic and festival food practices as forms of cultural preservation, community building, and emotional wellbeing. By focusing on Nepalese students in the U.S., it highlights the role of foodways in maintaining ethnic identity, religious traditions, and cultural continuity within a diasporic context, thereby extending dietary acculturation scholarship beyond nutrition and adaptation toward questions of cultural sustainability and belonging. Rather than passively adopting host culture norms, Nepalese international students demonstrated cultural resilience and agency by using ethnic and festival foods to maintain and adapt their traditions. This process, however, involved navigating various challenges such as time limitations, limited culinary skills, and restricted access to ingredients. While communal efforts and student networks supported traditional practices, adherence varied by individual capacity and resources. Food-related rituals were selectively maintained, often influenced by family and community ties. Emotional connections to traditional foods reinforced their infuence in cultural identity and wellbeing. Overall, Nepalese students' traditional food practices in the U.S. reflected a nuanced negotiation between cultural preservation and adaptation, illustrating how tradition endures, transforms, or pauses depending on context and capability.

This study underscores the central role of cultural foods, including ethnic and festival dishes, in preserving identity, fostering belonging, and supporting emotional wellbeing among international students. Despite this implication, the continuation and adaptation of such food practices remain underrepresented in the academic literature. Future research should address this gap to inform culturally responsive interventions that promote sustainable and healthy dietary behaviors among international student populations. Further studies are encouraged to examine how food-related cultural adaptation varies across factors such as geographic region, gender, and duration of residence abroad. Employing mixed-methods designs could yield a more comprehensive understanding of the complex interplay between cultural, psychological, and environmental influences on dietary acculturation. Longitudinal research would also be valuable in tracking the evolution of food practices over time and assessing the extent to which these adaptations persist post-graduation, potentially influencing long-term dietary habits and cultural identity integration.

Participants' narratives revealed the importance of social infrastructure such as peer networks, cultural organizations, and community support in maintaining traditional food practices. Based on these findings, Nepalese international students are encouraged to actively engage in social networking and communal initiatives to preserve and celebrate their cultural food traditions. Forming strong peer networks and participating in Nepalese student associations can offer valuable social support, ease feelings of isolation, and provide opportunities to collectively prepare and enjoy traditional meals, especially during festivals and cultural events. Organizing potlucks, cooking sessions, or cultural food nights through these associations not only strengthens community bonds but also reinforces cultural identity and emotional wellbeing. Students are also encouraged to take initiative in creating or joining online groups where they can share recipes and cooking tips of ethnic and festival foods.

The findings also suggest practical implications for universities and international student support services. Institutions should consider facilitating access to culturally relevant ingredients, providing communal cooking spaces, and partnering with cultural student organizations to support food-centered programming, particularly around major ethnic and religious festivals. Orientation materials and community guides that include information on local ethnic grocery stores, ingredient sourcing, and culturally relevant cooking workshops may assist students in navigating food environments. Moreover, interventions that build culinary skills such as peer-led cooking classes could empower students to continue preparing traditional dishes despite limited experience or access. Recognizing the role of food in emotional wellbeing, mental health professionals and student support staff should receive training to understand the cultural dimensions of food and integrate this awareness into wellness programming.

Finally, academic institutions should consider the timing of significant cultural and religious festivals in academic scheduling or, at minimum, encourage flexibility from instructors to accommodate cultural expression and observance. As this study demonstrates, the continuation of traditional food practices among Nepalese students in the U.S. reflects not only dietary preference but also cultural resilience and emotional adaptation. These findings contribute to broader discussions in food studies, migration, and higher education, and underscore the need for institutional policies and research that support the cultural continuity and wellbeing of international students.

## Data Availability

The raw data supporting the conclusions of this article will be made available by the authors, without undue reservation.
